# Supplementary material for: Are carbon emissions trading and green financial instruments synergistic? -Comprehensive quantitative research based on content analysis
Source: PLoS One. 2024 Mar 7;19(3):e0298601. doi: 10.1371/journal.pone.0298601 (PMC10919601; doi:10.1371/journal.pone.0298601)
Supplement: S1 Data — (ZIP) [file pone.0298601.s001.zip › Data/地级市Z_F工作报告.zip!/╕ú╜¿/╞╬╠∩╩╨/└┤╫╘╣1⁄2╓┌║┼í╛┬φ┐╦╩2╛▌═°í┐_2013.docx]

各位代表：

现在，我代表莆田市人民政府向大会报告工作，请予审议，并请市政协各位委员和其他列席同志提出意见。

持续发展、有效作为的2012年

过去的一年，在市委的坚强领导下，市政府团结和依靠全市人民，围绕海西建设战略部署，面对复杂严峻的宏观经济环境，牢牢把握稳中求进总基调，大力实施“以港兴市、产业强市”发展战略，加快推进湄洲湾港口城市建设，基本完成市六届人大一次会议确定的目标任务。

国民经济健康发展，产业结构持续优化。初步预计，实现地区生产总值1200亿元，增长12.8%。财政总收入129亿元，增长24.9%，税性比重达88.1%，其中，地方级收入77.4亿元，增长21.1%。金融机构本外币存款余额1080亿元，增长23%；贷款余额920亿元，增长23%。实现社会消费品零售总额375亿元，增长15%。第一产业实现增加值108亿元，增长3%。农林牧渔业总产值180亿元，增长3.3%。农业产业化水平不断提升，138家市级以上农业产业化龙头企业销售收入168亿元，增长15%。天怡现代农业在香港成功上市。出口蔬菜质量安全示范区荣获“国家级食品农产品质量安全示范区”称号。第二产业实现增加值699亿元，增长16%。按原口径，规模以上工业产值1808亿元，增长16.5%。工业增加值578亿元，增长14.5%，对经济增长贡献率达58%；建筑业增加值121亿元，增长24%。第三产业实现增加值393亿元，增长10%。三次产业比例由上年的9.4：58.4：32.2调整为9.0：58.2：32.8。

项目带动坚实有力，发展后劲持续增强。坚持项目为纲、投资为要，全社会固定资产投资943亿元，增长30%。392个重点项目完成投资656亿元，完成年计划的115.5%。“五大战役”均超额完成年度计划。“三维”项目成功对接246个，完成年度投资298亿元。鞍钢冷轧、仙游抽水蓄能电站首台机组、莆永高速公路莆田段等项目建成投产或投入运营；金钟水利枢纽工程、赛得利差别化纤维项目主厂房、向莆铁路莆田段路基工程、湄洲湾港口铁路支线陆域路段、沈海复线高速公路莆田段路基工程等项目基本完工；湄渝高速公路萩芦至五星段、乌溪水库等项目开工建设；一批项目前期工作取得突破，林浆纸一体化项目环评获得环保部审批，石门澳、涵江港海洋功能区划调整获国务院审批。

城乡建设统筹推进，宜居环境持续改观。新建、续建木兰大道一期等多条城市道路、里长约100公里；改建荔园路等多条城市道路、里长约20公里；“白改黑”路面约52.6万平方米。中心城区路网密度每平方公里达7公里，人均城市道路面积近14平方米。建成区面积达69.2平方公里，城镇化率达51%。万达广场建成开业。文献广场、木兰金融财富中心等一批城市综合体项目加快推进。新建 25个商住小区，约2万套、270万平方米。燃气管网铺设以及加气站建设、污水处理厂及配套管网等项目加快建设，城市生活垃圾焚烧发电厂二期竣工投入运营，城市垃圾处理率98.6%、污水处理率83.5%。城乡信息化基础网络初步建立。新建绶溪公园一期等城市公园，新增绿地面积120公顷，绿化覆盖率46%，荣获“国家园林城市”称号。城市管理志愿服务活动、中心城区施工围挡违法占道专项整治和道路交通安全综合整治“大会战”取得实效。全国节水型社会建设试点、创建全国绿化模范城市通过验收。湄洲岛被国务院批准为国家级风景名胜区，木兰陂等9个水利风景区荣获“省级水利风景区”称号。小城镇综合改革试点工作顺利推进。村庄环境综合整治、农村家园清洁行动深入开展。

改革开放纵深推进，发展活力持续激发。《莆田市城乡一体化综合配套改革试验总体方案》获省委、省政府批准并启动实施。深化行政审批制度改革和服务标准化管理，市级审批服务事项减少196项，即办事项比率提高197.6%，审批效率提高42.2%。市属八大集团公司得到整合提升，资产总额突破200亿元，增长40.8%。发行首支市属国有企业11亿元债券。企业“一套表”统计制度改革、城乡住户调查一体化改革、水务一体化改革顺利推进；林业、医药卫生、文化、社会保障体系等改革继续深化。荣获文化部国有文艺院团体制改革突出贡献奖。推行就医就诊、社会保障“一卡通”。新型农村社会养老保险137万人，参保率达90%以上；基本医疗保险参保率达95%以上；新农合参合人数260万人，参合率99.98%，居全省首位。基本完成全市交通综合行政执法改革。理顺城市管理机制。推进公共资源市场化配置，优化网上交易系统，进场交易项目974个，金额达68.8亿元，节约和增值资金10亿元。全省科技创新型城市试点工作积极开展，莆田高新区成功升级为国家级高新区，6家企业成立院士专家工作站，100家企业建立市级以上技术中心，专利授权量累计突破千件。福莆宁同城化工作有序开展，闽东北经济协作工作继续推进。与马来西亚诗巫市建立国际友好城市关系。成功举办第十四届中国·湄洲妈祖文化旅游节、第七届中国（莆田）海峡工艺品博览会、第四届南少林文化武术节、第四届海峡论坛·妈祖文化活动周，举行5场国（境）外重大招商活动。全年实际利用外资（验资口径）2.55亿美元，与上年基本持平；外贸出口29.5亿美元，增长6%。

公共事业全面发展，人民生活持续改善。木兰溪重点流域水环境污染整治等13项31件为民办实事项目基本完成。626所中小学初步实现标准化建设。校安工程完成一期项目建设。6所中职校入选全省首批“达标中等职业学校”。建成仙游师范新校区一期工程。莆田学院评估整改工作、湄洲湾职业技术学院创建省级示范性高职院校持续推进。《妈祖》电视剧完成拍摄并在央视播出。莆仙大剧院、乡镇文化站、农家书屋等一批文化惠民工程基本建成。市儿童医院、乡镇卫生院改扩建等一批医疗卫生项目加快建设，305个村卫生所初步实现标准化建设，药品配送覆盖所有行政村。成功举（承）办莆田南少林功夫环球争霸赛、第五届亚洲体操锦标赛、全国羽毛球锦标赛、第三届市运会等重要赛事。体育健身工程点覆盖所有行政村。完成全市低保对象核查评定，补助资金全额发放。实施城乡医疗救助14990人次，救助金额1105万元。市社会福利中心、市慈康医院二期主体工程基本完工。新增城镇就业2.11万人，完成省下达指标的105.5%。城镇登记失业率2%，低于2.5%以内的目标控制线。建成保障性安居工程7986套。住房公积金新增缴存单位693个，发放贷款7.1亿元。城镇居民人均可支配收入24750元，增长13.3%；农民人均纯收入10300元，增长13.6%。人事、编制、电业、气象、防震、保密、地方志、档案等工作得到加强。

社会管理不断创新，和谐局面持续巩固。新建市发展服务中心大楼和43个镇街便民服务中心。加强省外劳务协作，引进外来劳动力3.3万人。组织职业技能鉴定2.8万人。积极维护职工权益，农民工劳动合同签订率达94.2%，劳动争议受理案件结案率达98.3%。全面完成村（居）委会换届选举。成立市社会组织评估委员会，加强社会组织规范管理。完成流动人口动态监测抽样调查工作。落实人口计生工作责任制，出生人口政策符合率90.8%。校园绿色网室建设持续推进。客运市场、出租车市场、餐桌污染、食品药品安全、扫黄打非和打击侵犯知识产权等专项治理工作扎实开展。成功防御两场暴雨、四次台风。安全生产监管体系更加完善，各项控制指标全面下降。信访局面总体平稳。平安创建工作扎实推进，治安状况大为改观，社会保持和谐稳定。双拥工作不断深化，优抚安置标准得到提高。国防动员、民兵预备役、海防、消防、人防等工作取得新进展。支持工、青、妇等群团组织发挥桥梁纽带作用。老区、外事、贸促会、残联、民族宗教、侨务、红十字会等工作取得新进步。

自身建设不断加强，政府形象持续提升。积极建设学习型政府，依法行政理念得到强化。自觉接受人大及其常委会监督，认真执行人大决议、决定，支持人民政协履行政治协商、民主监督、参政议政职能，办理市人大代表议案建议237件、市政协提案291件，办结率均为100%。受理行政复议案件98件，办结率100%，有效化解行政争议。开通“12345”政务服务热线，电子政务一体化办公平台加快建设，政府网站服务功能整体提升。办事公开规范化、标准化、常态化建设扎实推进，加强对规范自由裁量权制度执行情况的后续监管，绩效管理工作、政风行风建设持续推进。严格执行预算，降低行政成本。落实公务车辆管理长效机制。推进廉政风险防控工作，行政监察和审计工作得到加强，惩治和预防腐败体系建设取得新进展。

各位代表，一年来的实践和成就，充分展现了全市干部群众勇于创新、敢于创先的顽强斗志；充分展现了社会各界齐心合力、同舟共济的协作精神；充分展现了莆阳大地天时、地利、人和的和谐风貌！在此，我代表莆田市人民政府，向全市人民致以崇高的敬意！向人大代表、政协委员、各民主党派、工商联、无党派人士、各人民团体和社会各界，向离退休老领导、老同志，向驻莆部队、武警官兵和公安民警，向央属和省属单位，向港澳台同胞、广大侨胞和海内外朋友，致以崇高的敬意和衷心的感谢！

在取得成就的同时，也要看到经济社会发展中仍然存在一些问题和困难，主要是：农业经济效益不高，工业规模不大，现代服务业发展较慢，经济结构仍需大力调整；产业龙头企业偏少，产业链延伸还不够，高新技术产业比重不高，自主创新能力还不强，转变经济发展方式的任务还十分艰巨；城乡公共基础设施配套不足，城郊脏乱差和城区交通不畅问题突出，城市综合管理仍需大力加强；城乡、区域发展还不协调，基本公共服务不均衡，教育、卫生、文化等社会事业还不能满足人民群众的需求，促进城乡发展一体化任务繁重；土地、资金、人才等要素制约比较突出，破解发展难题的办法有待进一步创新；政府职能和机关作风还需进一步转变，机关办事效率有待进一步提高，等等。对于这些问题，我们要勇于面对，主动化解，务求实效。

抢抓机遇、奋发进取的2013年

2013年是全面贯彻落实党的十八大精神的开局之年，是实施“十二五”规划承前启后的关键之年，也是推进莆田市城乡一体化综合配套改革试验的攻坚之年。我们要审时度势抢机遇，坚定信心促发展，勇于担当抓落实，促进经济社会科学发展、跨越发展。

政府工作的总体要求是：全面贯彻落实党的十八大精神和中央经济工作会议精神，继续把握稳中求进工作总基调，紧紧盯住“跨越发展、宜居港城”目标，深入实施“以港兴市、产业强市”发展战略，着力稳增长、转方式、调结构、扩内需，着力推进城乡一体化综合配套改革试验，着力拓展“五大战役”内涵，更加注重提高增长质量和效益，更加注重激发发展活力和动力，更加注重社会民生保障和改善，实现经济持续健康发展、社会和谐稳定，努力建设富裕莆田、和谐莆田、文化莆田、生态莆田和幸福莆田，为全面建成小康社会奠定坚实基础。

经济社会发展的主要预期目标是：全市生产总值增长12.5%，预期1370亿元，其中，第一、二、三产业增加值分别增长2.5%、15%和10.5%；规模以上工业产值增长16%，预期1980亿元；全社会固定资产投资额增长20%，预期1115亿元；社会消费品零售总额增长15%，预期431亿元；外贸进出口总额增长10%，预期48.5亿美元；按验资口径，实际利用外资3亿美元，增长18%；财政总收入增长18%，预期151亿元，其中，地方级收入增长16%，预期88亿元；城镇居民人均可支配收入增长12%左右，农民人均纯收入增长15%左右；居民消费价格总水平涨幅控制在3%左右；城镇登记失业率控制在3%以内；人口自然增长率控制在7.3‰以内；单位生产总值能耗降低、主要污染物排放减少等约束性指标完成省下达任务。

实现上述目标，必须做好以下几个方面工作：

（一）坚持统筹发展，推进城乡一体

调整优化城乡发展布局。全力推进城乡一体化综合配套改革试验工作，重点抓好产业发展、公共服务、基础设施、规划建设、社会管理等5大专项、170个分项改革任务。引导城乡资源要素合理流动，推动农业向规模经营集中、工业向园区集中、农民居住向城镇及规划中心村集中，促进城乡工业企业配套协调发展、城乡社会化服务、农业产业化和现代化发展一体化，加快形成城乡产业关联紧密、空间布局合理、资源深度整合、区域特色明显、基础设施共享的发展新格局。

开展幸福家园试点建设。整合扶持政策，以“美丽莆田·幸福家园”试点建设为突破口，以土地整理和村庄整治为切入点，开展首批“两片、八点”共16个村试点建设，抓好新型农村社区规划建设、确权发证、产权交易流转等重点任务，争取建成5个农村新型社区。实施“三通”、“四绿”和“十配套”工程，实行生活垃圾、生活污水无害化处理，促进基本公共服务均等化。

推进小城镇和新农村建设。继续推进一批基础设施、产业、城建等项目建设。综合整治各试点镇主要街道环境景观，提高绿化水平，加快污水、垃圾处理设施建设，切实改善镇容街貌。落实土地增减挂钩、“三旧”改造等小城镇试点建设优惠政策，敢于先行先试，持续创新工作机制，及时总结、推广先进经验。进一步推进城市基础设施向农村辐射延伸，继续推进扶贫开发、造福工程等新农村建设。

（二）注重以港兴市，发展海洋经济

完善港口设施。新建、续建秀屿、莆头、罗屿、东吴作业区20个深水泊位，国投湄洲湾煤炭码头一期等专用码头，以及湄洲湾三期航道等一批港航项目，提高湄洲湾北岸港口吞吐能力。推进涵江、北高作业区建设，启动涵江港建设，续建三江口作业区陆岛交通码头，加快建设兴化湾南岸港口基础设施。完成文甲陆岛交通码头改造工程和宫下旅游专用码头建设，续建东海救助局湄洲基地。完善港区通讯、供水、供电、堆场、仓储、锚地等公共服务配套设施，推进港口扩大开放。

建设疏港通道。建成向莆铁路莆田段、湄洲湾港口铁路东吴支线、福厦铁路仙游客运站工程，加快建设各铁路专用线。建成沈海复线高速公路莆田段，加快建设湄渝高速公路莆田段。建成赤港至三江口疏港公路一期工程，加快省道201、202、仙港大道路面工程、后海303路堤至黄石的兴化湾南岸大通道、石城作业区至新厝店、秀屿作业区至木材加工区、莆头作业区至秀屿互通等疏港公路建设，力争开工建设罗屿作业区至渡口疏港公路及东吴作业区至忠门互通疏港公路。加快宁海新桥建设。

壮大临港产业。加快铁矿石、木材、油品、天然气和煤炭“五行”基地建设。培育发展能源、石化、钢铁、浆纸、装备制造等临港产业。加快建设LNG产业园、国投湄洲湾（石门澳）产业园、仙港新兴产业园，提高港口腹地产业规划水平和承载能力。开工建设林浆纸一体化、丁基橡胶、盛达橡胶、厦航沥青、平海湾海上风电等项目。扎实推进神华煤电一体化、泰盛纸业、蓝海高新材料等重大项目前期工作。加快发展临港服务业，重点实施 “五行”物流基地发展战略，积极引进国际知名物流企业，推进东吴临港物流园、兴化湾南岸区域物流园、黄石商贸物流园、笏石物流园、仙作物流园、莆头木材集散基地、铁路港湾站等建设。积极发展海上运输业。加强与向莆铁路沿线城市的物流合作，构建港铁联运的现代物流体系。

发展海洋渔业。推进鲍鱼、花蛤、海带、龙须菜、石斑鱼等海产品繁育、生产基地建设，扩大“中国花蛤之乡”、“南日鲍地理标志”等特色品牌效应。结合人工渔礁项目、滨海大道、海上风电项目建设，推进平海湾、南日群岛海域立体化生态养殖示范基地建设，打造十八列岛海洋牧场，发展湾外深水养殖；积极扶持远洋捕捞业；重点建设中海源海洋生物高新技术产业园、后海垦区现代渔业产业园，组建南日岛海洋集团，培育一批海洋龙头企业和水产品精深加工产业。

（三）突出产业强市，优化产业结构

扩大现代农业效益。用好用足强农惠农富农政策，争取更多涉农项目列入国家、省级计划盘子，加快仙游县、秀屿区中央财政小型农田水利重点县建设，不断完善农业基础设施。培育农村新型集体经济、专业合作经济组织，引导各类社会资本参与现代农业建设。推进农业科技创新，加快基层农技推广补助项目建设。推进特色农业基地、特色农业品牌、特色农业产业建设，搞活农产品流通，支持利农集团等企业开展设施农业项目建设，扶持发展闽中有机等一批现代农业产业化龙头企业，推进农业产业化示范区建设，规划建设兴化湾南岸食品产业园，积极创建“省级农民创业园”。

做大工业经济规模。继续实施工业“双百工程”，着力推进“1213”行动计划。建成赛得利差别化纤维、福人木业、海峡纺织城、百利电子等30个计划投产项目；建设佳通轮胎技改、三棵树涂料二期、华峰新材料等70个在建项目；推进中锦石化、丙烷脱氢、核电等20个重大项目前期工作。继续理顺工业园区管理体制，出台扶持工业园区发展政策，重点推进工业园区基础设施和综合服务平台建设。实施“一业一策”、“一企一策”，促进鞋服、食品、林产等传统产业集群转型升级；做大做强中科中涵、中科华宇、威诺数控等一批重点骨干企业，大力发展战略性新兴产业；规划建设兴化湾南岸装备制造产业园，打造先进制造业产业集群。继续开展企业减负专项行动。鼓励企业争创品牌，支持双驰、沃特等企业改制上市。

提升第三产业比重。推进喜盈门建材家具广场、木兰金融财富中心、上塘珠宝城、福建海峡国际商贸城、文献（步行街）广场、中国古典工艺家具博览城等项目建设。加快建设专业市场，升级改造集贸市场，推动零售企业以直营连锁经营等方式发展社区便利店，并向农村延伸。鼓励网销企业自创品牌，培育壮大电子商务平台企业。规划建设安福网销商贸城。鼓励发展楼宇经济，加快建设西天尾等总部经济区，筹建莆田国际鞋业总部基地。积极引进、扶持发展中介服务业，以及信托、期货、金融租赁、汽车金融、企业财务公司、小额贷款公司等非银行金融机构。利用铁路站、高速公路出口，大力发展“道口经济”。加快物流信息平台建设，引进国际知名物流企业入驻莆田，引导企业内部物流社会化、专业化，推进海西区域性物流节点城市建设。加快发展旅游业，创新旅游项目开发和资源保护机制；完善旅游配套设施和公共服务；强化营销运作，扩大旅游品牌影响，壮大旅游市场主体；打造工艺美术等九大特色旅游产品，构建“南湄洲，北九鲤”的旅游业发展格局。

加快文化产业发展。注重挖掘和传承莆田历史文化。推进工艺美术城等六大文化产业园区建设，筹建莆阳历史文化创意产业园；加快发展创意设计等八大文化产业；扶持集友框业、欧雅艺术、华昌珠宝等重点企业发展，筹建国际油画销售总部基地，加强艺术精品创作生产和拍卖营销，不断提升文化产业发展水平。积极申报木雕古典家具国家级产品质检中心。争取承办更多国际性、全国性的体育赛事，发展壮大体育产业。大力弘扬妈祖文化，推进世界妈祖文化中心建设。办好第十五届妈祖文化旅游节、第八届海峡工艺品博览会、书画艺术节、古典工艺家具博览会等重要节会，组织“创意莆田”系列赛事，发展会展经济。

（四）拓展需求空间，拉动经济增长

保障投资较快增长。安排市重点项目432个，总投资8088亿元，年度计划投资700亿元。继续打好“五大战役”。加快建设以金钟水利枢纽引水配套工程、木兰溪防洪工程、乌溪水库工程等为重点的一批农林水利类项目，以仙游工艺创意产业园、海峡纺织城等为重点的一批产业类项目，以仙游抽水蓄能电站、国投湄洲湾火电厂等为重点的一批能源类项目，以妈祖城核心区、木兰大道等为重点的一批城建基础设施类项目，以“三馆一宫”等为重点的一批社会事业类项目，以国药莆田医药产业项目、莆田红木产业总部基地等为重点的一批商贸服务类项目。坚持领导挂钩重点项目、分级管理和督查联动制度，推进重点项目建设。

促进消费稳步增长。加强对市场秩序的整顿和规范，进一步整治虚假广告等损害消费者利益的现象，营造健康、安全、和谐的消费环境。拓宽消费者的投资渠道，促进居民资产收入的增长，夯实消费增长的物质基础。刺激即期消费需求，引导和拉动消费快速增长。促进消费升级，鼓励文化、旅游、健身、购房等消费，积极发展网络购物等新型消费业态，改变传统的消费观念，引导居民科学消费、可持续消费。

保持出口持续增长。进一步优化通关环境。对56家列入省政府 “一对一”重点扶持外贸出口企业，在政策扶持、人才引进、品牌创立、境外展销等方面予以倾斜，扶持企业做大做强。引导出口企业开展品牌培育、质量认证等工作，加快产品设计研发、国际营销服务等平台建设，提高出口产品国际竞争力。依托商务部授予的“莆田鞋类外贸转型升级专业型示范基地”，完善公共服务平台建设，提升企业网络销售能力。

（五）完善配套功能，提升城市品位

坚持规划先行。强化城乡一体化规划以及单元控规衔接，不断完善城乡规划。编制道路交通、水利、市政管线、能源场站、防灾等基础设施专项规划。结合地方特色和产业需要，编制特色重点片区和重点项目规划。全面完成集镇和村庄规划。健全规划工作机制，确保规划编制科学、执行有力、监督有效。

完善基础设施。加快主城区、滨海新城以及各县区城区建设。统筹推进新区开发与旧城改造，新建、续建壶公路一期等50条市政道路，完成324国道莆田城涵段路面“白改黑”工程，启动荔涵大道等5条道路“白改黑”工程，新增道路里程80多公里，改造提升面积50多万平方米。实施路灯远程监控及维护基地工程。新建一批城区停车场。新建香格里拉、喜来登等一批星级酒店。推进万科、永鸿等一批城市综合体建设。深入开展南北洋水系治理，推进中心城区防洪排涝整治。开工建设九华山植物园、囊山公园、西河公园等一批公园。加快建设中心城区荔枝林带绿道、湄洲大道等慢行步道，实施城际交界面和主要出入口生态绿化工程，促进城市环境、形象、内涵和品质的全面提升。

注重综合管理。完善电子政务数据中心建设，统筹全市政务数据资源，提高数字化城市管理水平。强化城市管理委员会职责，落实职能部门和辖区责任制，严格城市综合执法，以治理城区“脏、乱、差”为重点，以“六改四化二整治”为载体，把重心从主次干道延伸到背街小巷，从城区延伸到城乡结合部，突出抓好违章广告、占道经营、乱张贴、乱倒垃圾等专项执法。建立长效机制，遏制“两违”现象。继续推进道路交通安全综合整治 “三年行动”。开展市民文明素质教育实践活动以及文明城市、文明单位等创建活动，树立文明莆田的良好形象。

（六）加强创新合作，激发发展活力

统筹配套改革。重视发展县区域经济，鼎力支持仙游县快速发展，加大“老少边岛”扶持力度，促进区域协调发展。深化收入分配制度改革，努力提高城乡居民收入在国民收入分配中的比重。深化部门预算改革，强化财政资金监管，优化财政支出结构，保障财政刚性支出。完善财税征管和协税护税机制，积极涵养税源，营造宽严适度的税收环境。推行纳税评估，倡导诚信纳税。推进营业税改征增值税试点工作。发展资本市场，鼓励企业改制。防范金融风险，打击非法集资、高息放贷行为。鼓励金融机构创新金融产品和服务方式，加大对实体经济、小微企业、重点项目、现代农业和科技创新项目等贷款的投放力度，增加公共财政对民生项目的投入。严格控制机构编制，深入推进事业单位改革。加快户籍制度改革，放宽城镇户籍限制，吸纳农民有序转为城镇居民。完善征地（海）补偿机制，健全被征地（海）农民就业培训和社会保障制度。继续推进国有资产管理体制改革，提高国有企业投融资能力和经营水平。继续推进水利改革，建立农业水费财政直补机制。全面推进医疗卫生体制改革，健全两大市级医疗集团运作机制，鼓励社会办医。推行新农合与城镇居民医保制度的并轨统一、新农保与城镇居民养老保险的并轨统一。

加强科技创新。全面推进科技创新型城市试点建设，争创全国科技进步先进城市“五连冠”。实施“科技创新十大工程”，加快建设产业优化、园区建设、人才引进、要素聚集、成果转化等为重点的区域创新体系；加快建立以企业为主体、市场为导向、产学研相结合的技术创新体系和科技创新平台，鼓励企业建立技术研发中心，加快建设中科院海西研究院莆田中心，加大科普场馆和院士专家工作站建设力度。支持企业构建创新联盟。加强知识产权保护。以国家级标准进一步完善莆田高新区发展规划，重点发展电子信息、高端装备制造、新能源和新材料等产业，认定、扶持20家“科技小巨人”企业，整合、发挥莆田能工巧匠的传统优势，促进创新要素向企业集聚，“粗放工业”向“精细工业”转变，贴牌生产向品牌建设转变，增加“莆田创造”、“莆田出品”的正能量。

扩大合作交流。跟踪落实已签约的12个央企项目，跟进华电等8个在谈央企项目；跟踪落实差别化化学纤维二期等23个省重点外资项目，跟进28个投资超千万美元、20个投资超亿美元外资项目；争取新对接民营企业项目50项、总投资500亿元以上，切实提高签约项目履约率。抢抓新一轮世界产业调整和转移机遇，采取点对点、靶向式及小团组招商，加强与世界500强企业、行业领军企业和台港澳优势产业对接与合作。加快申报国家级台商投资区。扶持仙游台湾农民创业园发展壮大。配合做好福平莆宁城际轨道的前期工作，促进福莆宁同城化。进一步密切闽东北五市一区协作。不断拓展妈祖文化等海峡两岸民间文化交流内涵。加强与国际友好城市之间的合作交流。

（七）美化生态环境，提高宜居水平

强化节能减排。加大环保执法。强化对佳通轮胎等9家重点耗能企业管理，实施英博雪津等20个重点节能项目，做好中小锅炉煤改气工作，对年节约300吨及以上标准煤企业给予奖励，实现工业增加值能耗下降3.25%目标。加快闽中污水处理厂三期、仙游第二污水处理厂、钟山、游洋、江口、白沙污水处理厂及污水收集管网建设。推进湄洲湾火电厂脱硝工程改造。开展国土资源节约集约模范县区创建活动，加快低效建设用地二次开发，探索建立建设用地投入产出考核机制。继续推进畜禽养殖场减排。积极培育低碳产业。推进仙游等3个省级循环经济示范园区建设。

深化环境整治。继续实施城乡环境综合整治“点线面”攻坚计划。健全海陆一体化的海洋环境保护机制，加大截污工程建设和治理力度，加快荔城区全国中小河流治理重点县建设，推进近岸海域水环境治理，启动建设湄洲湾北岸尾水排海大通道工程。实施湄洲岛海岸线修复工程。加强东圳库区等饮用水源地达标整治和备用水源地建设，保障饮水安全。开展环境卫生、大气污染、城市噪音、农村河道和建筑运输扬尘等专项治理。扩大农村环境综合整治范围，深入开展公路沿线、农村社区、农业面源、畜禽养殖等综合整治，创建一批农村家园清洁示范乡镇。

加强生态保护。继续实施“四绿”工程，推进森林抚育工程。开展“绿色乡镇”创建工作。加大生态公益林、自然保护区、生态功能区和土海湿地、湄洲岛海洋公园以及生物多样性管护力度。积极创建木兰溪源国家级自然保护区。落实耕地保护制度。健全生态补偿机制。严格河道（海域）采砂、矿产资源开采监管。继续推进水土流失、“青山挂白”、工矿废弃地恢复治理和山体保护复绿。开展国家生态城市、森林城市、环保模范城市等生态文明创建活动。

（八）重视改善民生，促进社会和谐

发展社会事业。办好治理餐桌污染；整治东圳水库污染源；扶持畜禽、水产标准化养殖；建设2300套保障性住房；综合整治市容市貌；城涵河道综合整治及慢行工程建设；建设“六山”公园；建设博物馆、图书馆、科技馆、青少年宫；建设20个文化激情广场；新增公交汽车200辆、出租车100辆；新增4000个中小学学位；续建市医院主体病房；提高农村低保补助水平；残疾人康复扶贫助学安居工程；建设湄洲岛游客服务中心；建设网格化社区服务体系等16项为民实事。统筹城乡教育资源，加强教师队伍建设，实施素质教育，推进义务教育均衡化、标准化，职业教育适用化，普通高中多样化发展。继续实施特教提升、公办幼儿园建设、中小学校舍、校车安全等教育工程。支持莆田学院发展，扶持湄洲湾职业技术学院创省级示范性职业院校。筹建莆田网络电视教育频道和教育集团。深入实施人才强市战略。加快建设市档案馆、乡镇综合文化站、村居文化活动中心等文化设施，扩大公共文化基础设施覆盖面。加快有线电视数字化工程建设。加强对文物和非物质文化遗产的保护传承，推进莆仙戏、仙作技艺“申遗”工作。筹建市中医医院，推进市第一医院新门诊大楼、市妇幼保健院迁建、县级医院、乡镇卫生院改扩建、社区卫生服务中心、村卫生室等医疗卫生设施建设，强化城乡药品监管，努力实现基本公共卫生服务均等化。完善城乡体育设施建设，广泛开展全民健身活动，促进群众体育、竞技体育上新水平。坚持计划生育基本国策，全面实施“生育文明·幸福家庭”促进计划，提高出生人口素质，促进人口长期均衡发展。实施更加积极的就业、创业扶持政策，鼓励多渠道多形式就业，促进创业带动就业，稳定就业基本面。提高城乡低保补助水平。加快发展慈善、社会福利、老龄和残疾人事业，建成市社会福利院、市慈康医院。加大安置房建设力度。做好我市第三次全国经济普查工作。积极发展档案、侨务、气象、科普、地方志和公积金等社会事业。

创新社会管理。深化“数字莆田”建设，推进城市“一卡通”、视频监控共享等项目建设。建立健全信用体系，推进诚信社会建设。加强信息系统工程建设和文化市场监管。整顿网络秩序。加强社区物业管理，努力实现商住小区物业管理全覆盖，促进和谐社区建设。调整现行户口迁移政策，稳步推进进城务工人员市民化。探索建立“村级组织+小区自治”等农村新型社区管理模式。支持工会、共青团、妇联等人民团体依法开展工作，引导社会组织健康有序发展，发挥群众参与社会管理的基础作用。完善和创新流动人口和特殊人群管理服务。落实矛盾纠纷大调解机制，加大信访源头治理力度，严格执行信访路线图，畅通和规范群众诉求表达、利益协调、权益保障渠道。深化“平安莆田”建设，完善立体化社会治安防控体系，严密防范和严厉打击各类违法犯罪活动。严格落实安全生产“一岗双责”和企业主体责任，强化消防等公共安全体系和企业安全生产基础设施配套建设。完善应急管理工作机制，提高处置突发事件的能力。认真落实民族宗教政策。做好信息化条件下的保密工作。加强国防意识教育。认真落实拥军优属条例，进一步做好双拥工作。

改进作风 注重实干

建设务实高效清廉政府

各位代表，全面建成小康社会是全市人民的重托和期待！我们一定要牢记“人民政府”前面的“人民”二字，身体力行中央关于改进工作作风密切联系群众八项规定，始终把维护人民利益放在首位，夙夜在公，勤勉工作，努力赢得人民群众的信任和拥护。

注重学习与创新。打铁还需自身硬。面对“跨越发展、宜居港城”新形势、新任务、新要求，多向群众学习，多向实践学习，注重知识更新，优化知识结构，提高科学决策能力；多做调查研究，多谋利民之策，注重了解实情，创新工作方法，提高驾驭经济工作的能力，在学习中求创新，在创新中谋发展，在发展中增活力，做到解决困难有韧劲，谋划发展有思路，推进工作有激情。

注重服务与效率。牢固树立为经济发展服务、为社会公众服务、为企业和纳税人服务的施政理念，坚持寓管理于服务之中。 健全重大决策社会稳定风险评估机制，提高应对突发事件的能力。加强政风行风建设，减少会议、精简文件，切实改进文风会风。深入实际、深入基层、深入群众，讲实话、干实事，敢作为、勇担当，言必行、行必果。严惩推诿扯皮，强化行政问责，促进公务人员履职到位、尽责到位，提高机关办事效率和服务水平。

注重廉洁与守法。坚持标本兼治、综合治理、惩防并举、注重预防的方针，不断健全完善惩治和预防腐败体系。提高政府投资项目、预算执行情况、民生领域资金和公共财政使用绩效。厉行勤俭节约，反对铺张浪费，严格控制公务经费支出，力戒奢靡之风，规范机关事务管理。全面推进依法行政、依法治市，加强法制宣传培训，强化学法、尊法、守法意识，做到严格执法、文明执法，保证有法必依、执法必严、违法必究。深化信息公开、政务公开。自觉接受人大法律监督、政协民主监督、司法监督、社会监督和舆论监督，让权力在阳光下运行。

各位代表，站在科学发展的更高起点上，把握城乡一体化的改革机遇，我们将脚踏实地地担起历史赋予的重任，在党的十八大精神指引下，在中共莆田市委坚强领导下，凝聚力量，汇集智慧，攻坚克难，开拓创新，为全面建成小康社会而努力奋斗！

《政府工作报告》有关注解

1、三维：央企、民企、外企。

2、企业“一套表”统计制度：统一规范、方便企业填报的企业统计报表制度。

3、两片、八点：两片，上塘珠宝城片区和大地城——文甲片区；八点，榜头镇坝下村和紫泽村、黄石镇水南村、华亭镇涧口村、梧塘镇沁后村、埭头镇石城村和月塘乡东潘村、湄洲镇宫下村。

4、三通、四绿、十配套：三通，道路通、污水通、电讯通；四绿，绿色乡村、绿色屏障、绿色河岸、绿色通道；十配套，公园、小学、幼儿园、卫生服务站、文化站、社区超市、银行网点、村道路灯、公交停靠站、社区服务中心。

5、三旧改造：旧城镇、旧村庄、旧厂房的改造。

6、造福工程：对居住在生产生活条件恶劣地方的贫困群众实施搬迁的扶贫工程。

7、双百工程：100家重点工业企业（50家高成长型企业、50家行业龙头企业）和100个工业提升项目（70个重点技术改造项目、30个新投产项目）。

8、“1213”行动计划：推进兴化湾南岸机械装备产业园建设，培育20个战略性新兴产业企业，构筑10个企业服务平台，实施30项市级企业技术创新项目，加快产业转型升级步伐。

9、九大特色旅游：朝圣度假旅游，山水祈梦生态养生旅游，工艺美术文化旅游，红色生态观光旅游，禅武、禅茶、禅香文化旅游，莆田历史文化旅游，宜居莆田荔林水乡旅游，滨海休闲度假旅游，乡村旅游。

10、六改四化二整治：六改，主次干道“白改黑”工程、老旧小区和背街小巷改造工程、人行道板改造工程、架空电力通信缆线入地进管工程、主干道临街建筑物立面改造工程、污水收集并网改造工程；四化，美化、绿化、亮化、数字化；二整治，交通秩序大整治、市容环境大整治。

11、六大文化产业园区：莆田工艺美术城、海西文化创意产业城、仙游工艺产业园、上塘珠宝城、海西印刷包装与物流基地、莆田国际油画艺术产业园。

12、八大文化产业：文化旅游产业、工艺美术产业、创意设计产业、演艺娱乐产业、体育休闲产业、节庆会展产业、印刷发行产业、数字传媒产业。

13、科技创新十大工程：产业结构优化工程、企业创新主体工程、高新园区提升工程、院地合作深化工程、科技平台搭建工程、科技成果转化工程、知识产权创造工程、科技基地示范工程、创新人才激励工程、科技中介服务工程。

14、“科技小巨人”企业：主要从事高新技术及其产品的研制、开发、生产、经营和服务，有较强的技术创新能力和自主知识产权，有良好成长性和发展潜力，对相关产业发展将起到较大促进和引领作用的科技型中小企业。

15、精细工业：具有持续创新设计以及绝对高品质管控的高价值工业品的生产活动和生产体系。如瑞士的手表制造业。

16、“六山”公园：九华山休闲公园、壶公山休闲公园、天马山休闲公园、囊山休闲公园、凤凰山公园、大蜚山森林公园。

17、大调解：人民调解、司法调解和行政调解。

实现上述目标，必须做好以下几个方面工作：

（一）坚持统筹发展，推进城乡一体

调整优化城乡发展布局。全力推进城乡一体化综合配套改革试验工作，重点抓好产业发展、公共服务、基础设施、规划建设、社会管理等5大专项、170个分项改革任务。引导城乡资源要素合理流动，推动农业向规模经营集中、工业向园区集中、农民居住向城镇及规划中心村集中，促进城乡工业企业配套协调发展、城乡社会化服务、农业产业化和现代化发展一体化，加快形成城乡产业关联紧密、空间布局合理、资源深度整合、区域特色明显、基础设施共享的发展新格局。

开展幸福家园试点建设。整合扶持政策，以“美丽莆田·幸福家园”试点建设为突破口，以土地整理和村庄整治为切入点，开展首批“两片、八点”共16个村试点建设，抓好新型农村社区规划建设、确权发证、产权交易流转等重点任务，争取建成5个农村新型社区。实施“三通”、“四绿”和“十配套”工程，实行生活垃圾、生活污水无害化处理，促进基本公共服务均等化。

推进小城镇和新农村建设。继续推进一批基础设施、产业、城建等项目建设。综合整治各试点镇主要街道环境景观，提高绿化水平，加快污水、垃圾处理设施建设，切实改善镇容街貌。落实土地增减挂钩、“三旧”改造等小城镇试点建设优惠政策，敢于先行先试，持续创新工作机制，及时总结、推广先进经验。进一步推进城市基础设施向农村辐射延伸，继续推进扶贫开发、造福工程等新农村建设。

（二）注重以港兴市，发展海洋经济

完善港口设施。新建、续建秀屿、莆头、罗屿、东吴作业区20个深水泊位，国投湄洲湾煤炭码头一期等专用码头，以及湄洲湾三期航道等一批港航项目，提高湄洲湾北岸港口吞吐能力。推进涵江、北高作业区建设，启动涵江港建设，续建三江口作业区陆岛交通码头，加快建设兴化湾南岸港口基础设施。完成文甲陆岛交通码头改造工程和宫下旅游专用码头建设，续建东海救助局湄洲基地。完善港区通讯、供水、供电、堆场、仓储、锚地等公共服务配套设施，推进港口扩大开放。

建设疏港通道。建成向莆铁路莆田段、湄洲湾港口铁路东吴支线、福厦铁路仙游客运站工程，加快建设各铁路专用线。建成沈海复线高速公路莆田段，加快建设湄渝高速公路莆田段。建成赤港至三江口疏港公路一期工程，加快省道201、202、仙港大道路面工程、后海303路堤至黄石的兴化湾南岸大通道、石城作业区至新厝店、秀屿作业区至木材加工区、莆头作业区至秀屿互通等疏港公路建设，力争开工建设罗屿作业区至渡口疏港公路及东吴作业区至忠门互通疏港公路。加快宁海新桥建设。

壮大临港产业。加快铁矿石、木材、油品、天然气和煤炭“五行”基地建设。培育发展能源、石化、钢铁、浆纸、装备制造等临港产业。加快建设LNG产业园、国投湄洲湾（石门澳）产业园、仙港新兴产业园，提高港口腹地产业规划水平和承载能力。开工建设林浆纸一体化、丁基橡胶、盛达橡胶、厦航沥青、平海湾海上风电等项目。扎实推进神华煤电一体化、泰盛纸业、蓝海高新材料等重大项目前期工作。加快发展临港服务业，重点实施 “五行”物流基地发展战略，积极引进国际知名物流企业，推进东吴临港物流园、兴化湾南岸区域物流园、黄石商贸物流园、笏石物流园、仙作物流园、莆头木材集散基地、铁路港湾站等建设。积极发展海上运输业。加强与向莆铁路沿线城市的物流合作，构建港铁联运的现代物流体系。

发展海洋渔业。推进鲍鱼、花蛤、海带、龙须菜、石斑鱼等海产品繁育、生产基地建设，扩大“中国花蛤之乡”、“南日鲍地理标志”等特色品牌效应。结合人工渔礁项目、滨海大道、海上风电项目建设，推进平海湾、南日群岛海域立体化生态养殖示范基地建设，打造十八列岛海洋牧场，发展湾外深水养殖；积极扶持远洋捕捞业；重点建设中海源海洋生物高新技术产业园、后海垦区现代渔业产业园，组建南日岛海洋集团，培育一批海洋龙头企业和水产品精深加工产业。

（三）突出产业强市，优化产业结构

扩大现代农业效益。用好用足强农惠农富农政策，争取更多涉农项目列入国家、省级计划盘子，加快仙游县、秀屿区中央财政小型农田水利重点县建设，不断完善农业基础设施。培育农村新型集体经济、专业合作经济组织，引导各类社会资本参与现代农业建设。推进农业科技创新，加快基层农技推广补助项目建设。推进特色农业基地、特色农业品牌、特色农业产业建设，搞活农产品流通，支持利农集团等企业开展设施农业项目建设，扶持发展闽中有机等一批现代农业产业化龙头企业，推进农业产业化示范区建设，规划建设兴化湾南岸食品产业园，积极创建“省级农民创业园”。

做大工业经济规模。继续实施工业“双百工程”，着力推进“1213”行动计划。建成赛得利差别化纤维、福人木业、海峡纺织城、百利电子等30个计划投产项目；建设佳通轮胎技改、三棵树涂料二期、华峰新材料等70个在建项目；推进中锦石化、丙烷脱氢、核电等20个重大项目前期工作。继续理顺工业园区管理体制，出台扶持工业园区发展政策，重点推进工业园区基础设施和综合服务平台建设。实施“一业一策”、“一企一策”，促进鞋服、食品、林产等传统产业集群转型升级；做大做强中科中涵、中科华宇、威诺数控等一批重点骨干企业，大力发展战略性新兴产业；规划建设兴化湾南岸装备制造产业园，打造先进制造业产业集群。继续开展企业减负专项行动。鼓励企业争创品牌，支持双驰、沃特等企业改制上市。

提升第三产业比重。推进喜盈门建材家具广场、木兰金融财富中心、上塘珠宝城、福建海峡国际商贸城、文献（步行街）广场、中国古典工艺家具博览城等项目建设。加快建设专业市场，升级改造集贸市场，推动零售企业以直营连锁经营等方式发展社区便利店，并向农村延伸。鼓励网销企业自创品牌，培育壮大电子商务平台企业。规划建设安福网销商贸城。鼓励发展楼宇经济，加快建设西天尾等总部经济区，筹建莆田国际鞋业总部基地。积极引进、扶持发展中介服务业，以及信托、期货、金融租赁、汽车金融、企业财务公司、小额贷款公司等非银行金融机构。利用铁路站、高速公路出口，大力发展“道口经济”。加快物流信息平台建设，引进国际知名物流企业入驻莆田，引导企业内部物流社会化、专业化，推进海西区域性物流节点城市建设。加快发展旅游业，创新旅游项目开发和资源保护机制；完善旅游配套设施和公共服务；强化营销运作，扩大旅游品牌影响，壮大旅游市场主体；打造工艺美术等九大特色旅游产品，构建“南湄洲，北九鲤”的旅游业发展格局。

加快文化产业发展。注重挖掘和传承莆田历史文化。推进工艺美术城等六大文化产业园区建设，筹建莆阳历史文化创意产业园；加快发展创意设计等八大文化产业；扶持集友框业、欧雅艺术、华昌珠宝等重点企业发展，筹建国际油画销售总部基地，加强艺术精品创作生产和拍卖营销，不断提升文化产业发展水平。积极申报木雕古典家具国家级产品质检中心。争取承办更多国际性、全国性的体育赛事，发展壮大体育产业。大力弘扬妈祖文化，推进世界妈祖文化中心建设。办好第十五届妈祖文化旅游节、第八届海峡工艺品博览会、书画艺术节、古典工艺家具博览会等重要节会，组织“创意莆田”系列赛事，发展会展经济。

（四）拓展需求空间，拉动经济增长

保障投资较快增长。安排市重点项目432个，总投资8088亿元，年度计划投资700亿元。继续打好“五大战役”。加快建设以金钟水利枢纽引水配套工程、木兰溪防洪工程、乌溪水库工程等为重点的一批农林水利类项目，以仙游工艺创意产业园、海峡纺织城等为重点的一批产业类项目，以仙游抽水蓄能电站、国投湄洲湾火电厂等为重点的一批能源类项目，以妈祖城核心区、木兰大道等为重点的一批城建基础设施类项目，以“三馆一宫”等为重点的一批社会事业类项目，以国药莆田医药产业项目、莆田红木产业总部基地等为重点的一批商贸服务类项目。坚持领导挂钩重点项目、分级管理和督查联动制度，推进重点项目建设。

促进消费稳步增长。加强对市场秩序的整顿和规范，进一步整治虚假广告等损害消费者利益的现象，营造健康、安全、和谐的消费环境。拓宽消费者的投资渠道，促进居民资产收入的增长，夯实消费增长的物质基础。刺激即期消费需求，引导和拉动消费快速增长。促进消费升级，鼓励文化、旅游、健身、购房等消费，积极发展网络购物等新型消费业态，改变传统的消费观念，引导居民科学消费、可持续消费。

保持出口持续增长。进一步优化通关环境。对56家列入省政府 “一对一”重点扶持外贸出口企业，在政策扶持、人才引进、品牌创立、境外展销等方面予以倾斜，扶持企业做大做强。引导出口企业开展品牌培育、质量认证等工作，加快产品设计研发、国际营销服务等平台建设，提高出口产品国际竞争力。依托商务部授予的“莆田鞋类外贸转型升级专业型示范基地”，完善公共服务平台建设，提升企业网络销售能力。

（五）完善配套功能，提升城市品位

坚持规划先行。强化城乡一体化规划以及单元控规衔接，不断完善城乡规划。编制道路交通、水利、市政管线、能源场站、防灾等基础设施专项规划。结合地方特色和产业需要，编制特色重点片区和重点项目规划。全面完成集镇和村庄规划。健全规划工作机制，确保规划编制科学、执行有力、监督有效。

完善基础设施。加快主城区、滨海新城以及各县区城区建设。统筹推进新区开发与旧城改造，新建、续建壶公路一期等50条市政道路，完成324国道莆田城涵段路面“白改黑”工程，启动荔涵大道等5条道路“白改黑”工程，新增道路里程80多公里，改造提升面积50多万平方米。实施路灯远程监控及维护基地工程。新建一批城区停车场。新建香格里拉、喜来登等一批星级酒店。推进万科、永鸿等一批城市综合体建设。深入开展南北洋水系治理，推进中心城区防洪排涝整治。开工建设九华山植物园、囊山公园、西河公园等一批公园。加快建设中心城区荔枝林带绿道、湄洲大道等慢行步道，实施城际交界面和主要出入口生态绿化工程，促进城市环境、形象、内涵和品质的全面提升。

注重综合管理。完善电子政务数据中心建设，统筹全市政务数据资源，提高数字化城市管理水平。强化城市管理委员会职责，落实职能部门和辖区责任制，严格城市综合执法，以治理城区“脏、乱、差”为重点，以“六改四化二整治”为载体，把重心从主次干道延伸到背街小巷，从城区延伸到城乡结合部，突出抓好违章广告、占道经营、乱张贴、乱倒垃圾等专项执法。建立长效机制，遏制“两违”现象。继续推进道路交通安全综合整治 “三年行动”。开展市民文明素质教育实践活动以及文明城市、文明单位等创建活动，树立文明莆田的良好形象。

（六）加强创新合作，激发发展活力

统筹配套改革。重视发展县区域经济，鼎力支持仙游县快速发展，加大“老少边岛”扶持力度，促进区域协调发展。深化收入分配制度改革，努力提高城乡居民收入在国民收入分配中的比重。深化部门预算改革，强化财政资金监管，优化财政支出结构，保障财政刚性支出。完善财税征管和协税护税机制，积极涵养税源，营造宽严适度的税收环境。推行纳税评估，倡导诚信纳税。推进营业税改征增值税试点工作。发展资本市场，鼓励企业改制。防范金融风险，打击非法集资、高息放贷行为。鼓励金融机构创新金融产品和服务方式，加大对实体经济、小微企业、重点项目、现代农业和科技创新项目等贷款的投放力度，增加公共财政对民生项目的投入。严格控制机构编制，深入推进事业单位改革。加快户籍制度改革，放宽城镇户籍限制，吸纳农民有序转为城镇居民。完善征地（海）补偿机制，健全被征地（海）农民就业培训和社会保障制度。继续推进国有资产管理体制改革，提高国有企业投融资能力和经营水平。继续推进水利改革，建立农业水费财政直补机制。全面推进医疗卫生体制改革，健全两大市级医疗集团运作机制，鼓励社会办医。推行新农合与城镇居民医保制度的并轨统一、新农保与城镇居民养老保险的并轨统一。

加强科技创新。全面推进科技创新型城市试点建设，争创全国科技进步先进城市“五连冠”。实施“科技创新十大工程”，加快建设产业优化、园区建设、人才引进、要素聚集、成果转化等为重点的区域创新体系；加快建立以企业为主体、市场为导向、产学研相结合的技术创新体系和科技创新平台，鼓励企业建立技术研发中心，加快建设中科院海西研究院莆田中心，加大科普场馆和院士专家工作站建设力度。支持企业构建创新联盟。加强知识产权保护。以国家级标准进一步完善莆田高新区发展规划，重点发展电子信息、高端装备制造、新能源和新材料等产业，认定、扶持20家“科技小巨人”企业，整合、发挥莆田能工巧匠的传统优势，促进创新要素向企业集聚，“粗放工业”向“精细工业”转变，贴牌生产向品牌建设转变，增加“莆田创造”、“莆田出品”的正能量。

扩大合作交流。跟踪落实已签约的12个央企项目，跟进华电等8个在谈央企项目；跟踪落实差别化化学纤维二期等23个省重点外资项目，跟进28个投资超千万美元、20个投资超亿美元外资项目；争取新对接民营企业项目50项、总投资500亿元以上，切实提高签约项目履约率。抢抓新一轮世界产业调整和转移机遇，采取点对点、靶向式及小团组招商，加强与世界500强企业、行业领军企业和台港澳优势产业对接与合作。加快申报国家级台商投资区。扶持仙游台湾农民创业园发展壮大。配合做好福平莆宁城际轨道的前期工作，促进福莆宁同城化。进一步密切闽东北五市一区协作。不断拓展妈祖文化等海峡两岸民间文化交流内涵。加强与国际友好城市之间的合作交流。

（七）美化生态环境，提高宜居水平

强化节能减排。加大环保执法。强化对佳通轮胎等9家重点耗能企业管理，实施英博雪津等20个重点节能项目，做好中小锅炉煤改气工作，对年节约300吨及以上标准煤企业给予奖励，实现工业增加值能耗下降3.25%目标。加快闽中污水处理厂三期、仙游第二污水处理厂、钟山、游洋、江口、白沙污水处理厂及污水收集管网建设。推进湄洲湾火电厂脱硝工程改造。开展国土资源节约集约模范县区创建活动，加快低效建设用地二次开发，探索建立建设用地投入产出考核机制。继续推进畜禽养殖场减排。积极培育低碳产业。推进仙游等3个省级循环经济示范园区建设。

深化环境整治。继续实施城乡环境综合整治“点线面”攻坚计划。健全海陆一体化的海洋环境保护机制，加大截污工程建设和治理力度，加快荔城区全国中小河流治理重点县建设，推进近岸海域水环境治理，启动建设湄洲湾北岸尾水排海大通道工程。实施湄洲岛海岸线修复工程。加强东圳库区等饮用水源地达标整治和备用水源地建设，保障饮水安全。开展环境卫生、大气污染、城市噪音、农村河道和建筑运输扬尘等专项治理。扩大农村环境综合整治范围，深入开展公路沿线、农村社区、农业面源、畜禽养殖等综合整治，创建一批农村家园清洁示范乡镇。

加强生态保护。继续实施“四绿”工程，推进森林抚育工程。开展“绿色乡镇”创建工作。加大生态公益林、自然保护区、生态功能区和土海湿地、湄洲岛海洋公园以及生物多样性管护力度。积极创建木兰溪源国家级自然保护区。落实耕地保护制度。健全生态补偿机制。严格河道（海域）采砂、矿产资源开采监管。继续推进水土流失、“青山挂白”、工矿废弃地恢复治理和山体保护复绿。开展国家生态城市、森林城市、环保模范城市等生态文明创建活动。

（八）重视改善民生，促进社会和谐

发展社会事业。办好治理餐桌污染；整治东圳水库污染源；扶持畜禽、水产标准化养殖；建设2300套保障性住房；综合整治市容市貌；城涵河道综合整治及慢行工程建设；建设“六山”公园；建设博物馆、图书馆、科技馆、青少年宫；建设20个文化激情广场；新增公交汽车200辆、出租车100辆；新增4000个中小学学位；续建市医院主体病房；提高农村低保补助水平；残疾人康复扶贫助学安居工程；建设湄洲岛游客服务中心；建设网格化社区服务体系等16项为民实事。统筹城乡教育资源，加强教师队伍建设，实施素质教育，推进义务教育均衡化、标准化，职业教育适用化，普通高中多样化发展。继续实施特教提升、公办幼儿园建设、中小学校舍、校车安全等教育工程。支持莆田学院发展，扶持湄洲湾职业技术学院创省级示范性职业院校。筹建莆田网络电视教育频道和教育集团。深入实施人才强市战略。加快建设市档案馆、乡镇综合文化站、村居文化活动中心等文化设施，扩大公共文化基础设施覆盖面。加快有线电视数字化工程建设。加强对文物和非物质文化遗产的保护传承，推进莆仙戏、仙作技艺“申遗”工作。筹建市中医医院，推进市第一医院新门诊大楼、市妇幼保健院迁建、县级医院、乡镇卫生院改扩建、社区卫生服务中心、村卫生室等医疗卫生设施建设，强化城乡药品监管，努力实现基本公共卫生服务均等化。完善城乡体育设施建设，广泛开展全民健身活动，促进群众体育、竞技体育上新水平。坚持计划生育基本国策，全面实施“生育文明·幸福家庭”促进计划，提高出生人口素质，促进人口长期均衡发展。实施更加积极的就业、创业扶持政策，鼓励多渠道多形式就业，促进创业带动就业，稳定就业基本面。提高城乡低保补助水平。加快发展慈善、社会福利、老龄和残疾人事业，建成市社会福利院、市慈康医院。加大安置房建设力度。做好我市第三次全国经济普查工作。积极发展档案、侨务、气象、科普、地方志和公积金等社会事业。

创新社会管理。深化“数字莆田”建设，推进城市“一卡通”、视频监控共享等项目建设。建立健全信用体系，推进诚信社会建设。加强信息系统工程建设和文化市场监管。整顿网络秩序。加强社区物业管理，努力实现商住小区物业管理全覆盖，促进和谐社区建设。调整现行户口迁移政策，稳步推进进城务工人员市民化。探索建立“村级组织+小区自治”等农村新型社区管理模式。支持工会、共青团、妇联等人民团体依法开展工作，引导社会组织健康有序发展，发挥群众参与社会管理的基础作用。完善和创新流动人口和特殊人群管理服务。落实矛盾纠纷大调解机制，加大信访源头治理力度，严格执行信访路线图，畅通和规范群众诉求表达、利益协调、权益保障渠道。深化“平安莆田”建设，完善立体化社会治安防控体系，严密防范和严厉打击各类违法犯罪活动。严格落实安全生产“一岗双责”和企业主体责任，强化消防等公共安全体系和企业安全生产基础设施配套建设。完善应急管理工作机制，提高处置突发事件的能力。认真落实民族宗教政策。做好信息化条件下的保密工作。加强国防意识教育。认真落实拥军优属条例，进一步做好双拥工作。

改进作风 注重实干

建设务实高效清廉政府

各位代表，全面建成小康社会是全市人民的重托和期待！我们一定要牢记“人民政府”前面的“人民”二字，身体力行中央关于改进工作作风密切联系群众八项规定，始终把维护人民利益放在首位，夙夜在公，勤勉工作，努力赢得人民群众的信任和拥护。

注重学习与创新。打铁还需自身硬。面对“跨越发展、宜居港城”新形势、新任务、新要求，多向群众学习，多向实践学习，注重知识更新，优化知识结构，提高科学决策能力；多做调查研究，多谋利民之策，注重了解实情，创新工作方法，提高驾驭经济工作的能力，在学习中求创新，在创新中谋发展，在发展中增活力，做到解决困难有韧劲，谋划发展有思路，推进工作有激情。

注重服务与效率。牢固树立为经济发展服务、为社会公众服务、为企业和纳税人服务的施政理念，坚持寓管理于服务之中。 健全重大决策社会稳定风险评估机制，提高应对突发事件的能力。加强政风行风建设，减少会议、精简文件，切实改进文风会风。深入实际、深入基层、深入群众，讲实话、干实事，敢作为、勇担当，言必行、行必果。严惩推诿扯皮，强化行政问责，促进公务人员履职到位、尽责到位，提高机关办事效率和服务水平。

注重廉洁与守法。坚持标本兼治、综合治理、惩防并举、注重预防的方针，不断健全完善惩治和预防腐败体系。提高政府投资项目、预算执行情况、民生领域资金和公共财政使用绩效。厉行勤俭节约，反对铺张浪费，严格控制公务经费支出，力戒奢靡之风，规范机关事务管理。全面推进依法行政、依法治市，加强法制宣传培训，强化学法、尊法、守法意识，做到严格执法、文明执法，保证有法必依、执法必严、违法必究。深化信息公开、政务公开。自觉接受人大法律监督、政协民主监督、司法监督、社会监督和舆论监督，让权力在阳光下运行。

各位代表，站在科学发展的更高起点上，把握城乡一体化的改革机遇，我们将脚踏实地地担起历史赋予的重任，在党的十八大精神指引下，在中共莆田市委坚强领导下，凝聚力量，汇集智慧，攻坚克难，开拓创新，为全面建成小康社会而努力奋斗！
